# Differences in service and antibiotics use following symptomatic respiratory tract infections between 2016 and 2021 in rural Anhui, China

**DOI:** 10.1017/S0950268822000942

**Published:** 2022-05-25

**Authors:** Xiuze Xu, Kexin Zhang, Huan Ma, Xingrong Shen, Jing Chai, Mengsha Tang, Yanan Du, Qun Xue, Xiaoqin Guan, Guocheng Li, Debin Wang

**Affiliations:** 1 School of Health Service Management, Anhui Medical University, Meishan Road 81, Hefei, Anhui 230032, China; 2 School of Public Health, Anhui Medical University, Hefei, Anhui, China

**Keywords:** Antibiotic resistance, China, COVID-19, primary care, respiratory tract infections

## Abstract

In the past 10–15 years, the government of China has made various efforts in tackling excessive antibiotics use. Yet, little is known about their effects at rural primary care settings. This study aimed to determine the impact of government policies and the COVID-19 pandemic on antibiotic prescribing practices at such settings utilizing data from separate studies carried out pre- and during the pandemic, in 2016 and 2021 in Anhui province, China, using identical sampling and survey approaches. Data on antibiotics prescribed, diagnosis, socio-demographic, etc., were obtained through non-participative observation and a structured exit survey. Data analysis comprised mainly descriptive comparisons of 1153 and 762 patients with respiratory infections recruited in 2016 and 2021, respectively. The overall antibiotics prescription rate decreased from 89.6% in 2016 to 69.1% in 2021, and the proportion of prescriptions for two or more classes of antibiotics was estimated as 35.9% in 2016 and 11.0% in 2021. There was a statistically significant decrease in the number of days from symptom onset to clinic visits between the year groups. In conclusion, measures to constrain excessive prescription of antibiotics have led to some improvements at the rural primary care level, and the COVID-19 pandemic has had varying effects on antibiotic use.

## Introduction

Antimicrobial resistance (AMR) is regarded as a major public health problem of global concern and a significant threat to the clinical effectiveness of antibiotics [1, 2]. Globally, estimated deaths due to AMR infections have already reached 700 000 annually, and may exceed 10 million by 2050; the economic impact on the global economy is forecast to result in losses of USD 100 trillion, if this trend is not adequately contained [3]. The worldwide use of antibiotics has been reported to have increased by 39% between the years 2000 and 2015, with the major use in primary healthcare institutions [4, 5]. A 2015 study in China estimated that the direct economic loss due to overuse of antibiotics ranged from 92.55 to 98.93 billion RMB and an indirect loss from 17.37 billion to 18.12 billion RMB [6]. In the same period, consumption of antimicrobials in China increased at a rate of 82.6% from 2.3 to 4.2 billion cumulative defined daily doses [7]. Moreover, a cross-sectional study of 40 counties in rural western China reported that 48.4% of all service episodes at village clinics used at least one type of antibiotics [8]. In general, respiratory tract infections (RTIs) account for most primary healthcare visits, but relatively few cases prove to be due to bacterial infections [9]. However, approximately 70–90% of symptomatic RTI patients visiting village clinics were prescribed antibiotics [8, 10].

The Chinese government in the past decade has introduced a series of measures to stem excessive use of antibiotics which include: (i) reformation of the nationwide rural cooperative medical insurance system and implementation of zero-markup for prescribed medications [11]; (ii) introduction of national AMR and antibiotic use surveillance networks in 2005 [12]; (iii) development of lists of conditions which do not require treatment with antibiotics; and (iv) enactment of a comprehensive Special Antimicrobial Use Rectification Program in 2012 [13, 14]. Clearly defined antibiotic protocols to guide empirical therapy are available but need to be reinforced via regular education of, and feedback to, all doctors. As a result, the latest evidence suggests that antibiotic prescription rates at public and county or higher-level hospitals are decreasing [15, 16]. However, due to incomplete medical records, there is a general lack of information about antibiotic usage at rural village clinics, and limited studies indicate that antibiotic prescription rates remain very high [17, 18]. These include our earlier study in 2016, which documented rates as high as 80% for symptomatic RTI patients at village clinics in Anhui province [10]. In 2021, to evaluate an intervention package informed by our previous studies, we carried out another survey of antibiotic usage at the same type of clinic settings in Anhui using identical methods.

This paper uses data from the foregoing two studies and compares differences in service and antibiotics use following symptomatic RTIs with particular attention being paid to the number of days between symptoms onset and clinic visit (NDOV), the number of types of antibiotics used, the classes of antibiotics prescribed and their modes of administration. The NDOV index was used since delaying service and antibiotics use among RTI patients has become a proven strategy in containing bacterial resistance [19, 22]. Also, our previous studies suggest that the opportunity for RTI patients to visit clinics and receive prescribed antibiotics reduced sharply after 2–3 days following onset of infection [20]. Likewise, the greater the number of antimicrobials prescribed increased the potential for multi-resistance [21].

Between the two study years, China had not only witnessed various structural changes in relation to antibiotics use but also the intervention of the COVID-19 pandemic. All practicing physicians were held responsible for identifying, recording and referring suspected COVID-19 cases, and all symptomatic RTI cases along with medications for fever, cough and other associated symptoms were required to be entered into a surveillance network.

## Methods

### Sites and participants

The study used data from two separate projects funded by the UK-China Strategic Prosperity Fund and National Natural Science Foundation of China respectively. Participants were recruited using a stratified-cluster randomised sampling approach targeting RTI patients at rural primary care settings of Anhui province, and used similar sampling, observation and survey approaches [20, 23]. Selection of village clinics proceeded as follows: (i) all cities in the province were divided into north, middle and southern regions; (ii) five cities were randomly selected from each of the three regions, and four village clinics were designated from each of the cities; (iii) a village doctor from each clinic made initial observations and then recruited patients for at least 2 weeks according to preset inclusion/exclusion criteria. The patients were to be (i) males and females aged 18 years or older, (ii) able to consent to interview, (iii) having a symptomatic RTI and (iv) presenting as an outpatient for the first time within the study period.

### Data collection

Data were collected via direct non-participative observation and structured questionnaire exit surveys. The observation used a semi-structured survey questionnaire which detailed basic demographic and clinical information including presenting symptoms (as described by the patient), and physician diagnosis (as communicated to the patient). Patients meeting the inclusion criteria were followed-up through an exit survey before leaving the clinic. Data collected included socio-demographics, the NDOV index and the names of antibiotics prescribed. The proforma and exit questionnaire were designed and administered face to face by a researcher. Data collection for the first project started from 30 April 2016 and ended on 12 May 2016; the second was from 8 January 2021 to 18 June 2021.

### Outcome measures

The study used four outcome measures, i.e. (i) service and antibiotics use following symptomatic RTIs; (ii) the NDOV index; (iii) the number of types of antibiotics listed in single prescriptions and classes of antibiotics prescribed; and (iv) modes of antibiotics administration.

### Data analysis

Completed paper questionnaires were double entered into a database using EPIDATA V.3.1 and then extracted and analysed using SPSS V.26.0 and Microsoft Excel 2019. Cases with missing data were excluded. The analysis compared differences in the study periods between patient groups. Differences between groups were estimated using the *χ*^2^ test, and a *P* value < 0.05 indicated statistical significance.

## Results

### Socio-demographic characteristics

In this study, 1498 patients in 2016 and 939 in 2021 were observed. In the first period, 1153 met the inclusion criteria and 1072 completed both questionnaires; for 2021, the corresponding numbers were 762 and 702 respectively. As shown in Table 1, statistically significant differences were evident between the two study groups in terms of sex, age and diagnosis. Marginally higher numbers of females than males (57.5% *vs.* 42.5%) were recorded in 2021 which was largely similar in 2016 (51.0% *vs.* 49.0%). Participants in 2021 were slightly older than in the earlier year group (55 *vs.* 52 years). Most patients in both groups presented with an upper (U)RTI but there was a substantial increase (27%) in the 2021 over the 2016 group.

**Table 1.** Socio-demographics of patients with respiratory tract infections recruited in 2016 and 2021

Subgroups	2016	2021	*P*
Sex			
Male	547(51.0)	298(42.5)	<0.001
Female	525(49.0)	403(57.5)	
Age			
≤40	289(27.0)	159(22.8)	<0.001
41–50	198(18.5)	97(13.7)	
51–60	204(19.1)	202(29.0)	
61–70	245(22.9)	123(17.7)	
≥71	134(12.5)	115(16.5)	
Education			
Illiteracy	277(26.0)	211(30.1)	0.152
Primary school	352(33.1)	211(30.1)	
Middle school	304(28.5)	207(29.5)	
Higher school	132(12.4)	73(10.4)	
Diagnosis			
URTI	574(53.5)	567(80.8)	<0.001
LRTI	326(30.4)	85(12.1)	
Not clear	172(16.0)	50(7.1)	
Total	1072(100)	702（100）	

URTI, upper respiratory tract infection; LRTI, lower respiratory tract infection; Not clear, diagnosis unclear; (*N*/%).

### Number of days between symptom onset and clinic visit

Table 2 presents the differences in NDOV indices by different patient groups. All values between the two study groups proved statistically significant, and there was a consistent decrease in this index in all subgroups in both years. In general, male patients, those ≤40 and ≥71 years, the higher educated, those with fewer symptoms and not diagnosed as having an URTI, tended to consult village doctors earlier in 2021 than 2016. Overall, visits to clinics most often peaked on the second day of onset for all the symptoms recorded in 2016 but notably so for fever and discomfort on the first day in 2021.

**Table 2.** Difference in number of days between symptom onset and visit to clinic by different patient groups

Subgroups	2016				2021				*P*
	1–2	3–4	5–7	>7	1–2	3–4	5–7	>7	
Sex									
Male	167(30.5)	169(30.9)	106(19.4)	105(19.2)	169(56.7)	76(25.5)	27(9.1)	26(8.7)	<0.001
Female	175(33.3)	149(28.4)	101(19.2)	100(19.0)	188(46.7)	113(28.0)	60(14.9)	42(10.4)	<0.001
*P*	0.744				0.029				
Age									
≤40	107(37.0)	99(34.3)	45(15.6)	38(13.1)	91(57.2)	41(25.8)	21(13.2)	6(3.8)	<0.001
41–50	73(36.9)	63(31.8)	36(18.2)	26(13.1)	51(52.6)	23(23.7)	13(13.4)	10(10.3)	0.085
51–60	63(30.9)	57(27.9)	41(20.1)	43(21.1)	101(50.0)	63(31.2)	23(11.4)	15(7.4)	<0.001
61–70	65(26.5)	65(26.5)	52(21.2)	63(25.7)	56(45.5)	32(26.0)	19(15.4)	16(13.0)	0.001
≥71	33(24.6)	33(24.6)	33(24.6)	35(26.1)	55(47.8)	29(25.2)	10(8.7)	21(18.3)	<0.001
*P*	<0.001				0.023				
Education									
Illiteracy	79(28.5)	67(24.2)	61(22.0)	70(25.3)	108(51.2)	60(28.4)	22(10.4)	21(10.0)	<0.001
Primary school	96(27.3)	116(33.0)	69(19.6)	71(20.2)	97(46.0)	64(30.3)	25(11.8)	25(11.8)	<0.001
Middle school	112(36.8)	95(31.3)	49(16.1)	48(15.8)	105(50.7)	51(24.6)	32(15.5)	19(9.2)	0.009
Higher school	53(40.2)	36(27.3)	28(21.2)	15(11.4)	48(65.8)	14(19.2)	8(11.0)	3(4.1)	0.004
*P*	<0.001				0.157				
Number of symptoms									
1	36(35.6)	30(29.7)	17(16.8)	18(17.8)	118(61.8)	45(23.6)	16(8.4)	12(6.3)	<0.001
2	101(35.9)	88(31.3)	43(15.3)	49(17.4)	122(53.0)	55(23.9)	31(13.5)	22(9.6)	0.001
3	101(28.9)	95(27.1)	80(22.9)	74(21.1)	69(42.1)	53(32.3)	23(14.0)	19(11.6)	0.001
>3	104(30.6)	105(30.9)	67(19.7)	64(18.8)	49(41.9)	36(30.8)	17(14.5)	15(12.8)	0.094
*P*	0.312				0.015				
Diagnosis									
URTI	235(40.9)	190(33.1)	78(13.6)	71(12.4)	297(52.4)	148(26.1)	73(12.9)	49(8.6)	0.001
LRTI	64(19.6)	86(26.4)	92(28.2)	84(25.8)	33(38.8)	26(30.6)	10(11.8)	16(18.8)	<0.001
Not clear	43(25.0)	42(24.4)	37(21.5)	50(29.1)	28(56.0)	15(30.0)	4(8.0)	3(6.0)	<0.001
*P*	<0.001				0.039				
Total	342(31.9)	318(29.7)	207(19.3)	205(19.1)	358(51.0)	189(26.9)	87(12.4)	68(9.7)	<0.001

### Number of types, classes and mode of administration of prescribed antibiotics between patient groups

The overall antibiotics prescription rate decreased from 89.6% in 2016 to 69.1% in 2021. The largest number of different antibiotics within a single prescription was four in 2016 and three in 2021, and the proportion of those receiving treatment with two or more types was estimated as 35.9% in 2016 and 11.0% in 2021. In terms of antibiotics class (Table 3), cephalosporins and penicillins were the most prescribed in both study periods, and cephalosporin usage remained unchanged at 33.7% and 32.2%, respectively; prescription rates for the other three classes of agents all decreased significantly. In 2016, statistically significant differences were evident between (i) sex- and age-subgroups for diagnosis of RTI, (ii) prescriptions of quinolones and cephalosporins and (iii) RTI diagnosis and quinolones and penicillin usage. Most of these differences disappeared in 2021, except for quinolone prescriptions between males and females.

**Table 3.** Difference in classes of prescribed antibiotics by different patient groups

Subgroups	2016				2021				*P*			
	Quinolones	Cephalosporins	Penicillins	Others	Quinolones	Cephalosporins	Penicillins	Others	*P*_1_	*P*_2_	*P*_3_	*P*_4_
Sex												
Male	193(35.3)	200(36.6)	200(36.6)	37(6.8)	33(11.1)	89(29.9)	71(23.8)	30(10.1)	<0.001	0.050	<0.001	0.090
Female	144(27.4)	161(30.7)	204(38.9)	27(5.1)	26(6.5)	136(33.7)	82(20.3)	41(10.2)	<0.001	0.319	<0.001	0.004
*P*	0.006	0.041	0.438	0.263	0.029	0.277	0.237	0.963				
Age												
≤40	62(21.5)	87(30.1)	100(34.6)	22(7.6)	9(5.7)	57(35.8)	37(23.3)	9(5.7)	<0.001	0.213	0.013	0.436
41–50	62(31.3)	61(30.8)	78(39.4)	19(9.6)	5(5.2)	30(30.9)	25(25.8)	11(11.3)	<0.001	0.983	0.021	0.641
51–60	69(33.8)	67(32.8)	79(38.7)	9(4.4)	22(10.9)	59(29.2)	37(18.3)	25(12.4)	<0.001	0.429	<0.001	0.004
61–70	101(41.2)	79(32.2)	104(42.4)	8(3.3)	9(7.3)	41(33.3)	33(26.8)	15(12.2)	<0.001	0.834	0.003	0.001
≥71	43(32.1)	67(50.1)	42(31.3)	5(3.7)	12(10.4)	36(31.3)	19(16.5)	11(9.6)	<0.001	0.003	0.007	0.061
*P*	<0.001	0.001	0.187	0.022	0.249	0.739	0.221	0.254				
Education												
Illiteracy	92(33.2)	109(39.4)	106(38.3)	9(3.2)	19(9.0)	59(28.0)	45(21.3)	30(14.2)	<0.001	0.009	<0.001	<0.001
Primary school	116(33.0)	112(31.8)	145(41.2)	17(4.8)	18(8.5)	74(35.1)	43(20.4)	18(8.5)	<0.001	0.427	<0.001	0.078
Middle school	84(27.6)	92(30.3)	108(35.5)	29(9.5)	17(8.2)	68(32.9)	46(22.2)	15(7.2)	<0.001	0.536	<0.001	0.364
Higher school	43(32.6)	46(34.8)	43(32.6)	9(6.8)	5(6.8)	25(34.2)	19(26.0)	8(11.0)	<0.001	0.931	0.328	0.303
*P*	0.407	0.102	0.267	0.009	0.952	0.438	0.792	0.093				
Diagnosis												
URTI	159(27.7)	179(31.2)	224(39.0)	38(6.6)	45(7.9)	184(32.5)	126(22.2)	59(10.4)	<0.001	0.646	<0.001	0.022
LRTI	142(43.6)	114(35.0)	143(43.9)	9(2.8)	13(15.3)	32(37.6)	19(22.4)	10(11.8)	<0.001	0.646	<0.001	<0.001
Not clear	36(20.9)	68(39.5)	37(21.5)	17(9.9)	1(2.0)	10(20.0)	8(16)	2(4.0)	0.002	0.011	0.393	0.191
*P*	<0.001	0.106	<0.001	0.004	0.018	0.101	0.588	0.307				
Total	337(31.4)	361(33.7)	404(37.7)	64(6.0)	59(8.4)	226(32.2)	153(21.8)	71(10.1)	<0.001	0.517	<0.001	0.001

Others: other antibiotics except quinolones, cephalosporins and penicillins; *p*_1_ < 0.05 statistically significant in the prescription rate of quinolones between 2016 and 2021.

About one-half to one-third of prescribed antibiotics were administered intravenously (49.3% in 2016 and 31.7% in 2021, Table 4). Almost all usage by patient subgroups proved statistically significant except for subjects with higher school education and no clear diagnosis. In the earlier study group, intravenous antibiotic use tended to increase with age (43.7% ≤40 years and 51.7% ≥71 years) and patients with URTI were less likely to receive intravenous antibiotics.

**Table 4.** Difference in modes of antibiotic administration by different patient groups

Subgroups	2016			2021			*P*
	Oral	Intravenous	Intravenous and oral	Oral	Intravenous	Intravenous and oral	
Sex							
Male	239(49.5)	165(34.2)	79(16.4)	138(67.6)	63(30.9)	3(1.5)	<0.001
Female	237(52.0)	165(36.2)	54(11.8)	193(68.9)	83(29.6)	4(1.4)	<0.001
*P*	0.140			0.956			
Age							
≤40	139(56.3)	84(34.0)	24(9.7)	81(69.8)	34(29.3)	1(0.9)	0.003
41–50	100(56.2)	57(32.0)	21(11.8)	51(71.8)	18(25.4)	2(2.8)	0.027
51–60	75(42.6)	70(39.8)	31(17.6)	92(68.7)	41(30.6)	1(0.7)	<0.001
61–70	104(48.1)	69(31.9)	43(19.9)	56(65.1)	28(32.6)	2(2.3)	<0.001
≥71	58(48.3)	48(40.0)	14(11.7)	48(66.7)	23(31.9)	1(1.4)	0.008
*P*	0.012			0.919			
Education							
Illiteracy	114(46.5)	94(38.4)	37(15.1)	87(60.4)	54(37.5)	3(2.1)	<0.001
Primary school	170(54.8)	92(29.7)	48(15.5)	96(66.2)	48(33.1)	1(0.7)	<0.001
Middle school	127(48.5)	103(39.3)	32(12.2)	109(77.9)	30(21.4)	1(0.7)	<0.001
Higher school	62(53.4)	39(33.6)	15(12.9)	39(69.9)	15(26.8)	2(3.6)	0.061
*P*	0.210			0.039			
Diagnosis							
URTI	299(58.7)	159(31.2)	51(10.0)	272(70.6)	109(28.3)	4(1.0)	<0.001
LRTI	117(38.5)	110(36.2)	77(25.3)	43(60.6)	26(36.6)	2(2.8)	<0.001
Not clear	60(47.6)	61(48.4)	5(4.0)	16(55.2)	12(41.4)	1(3.4)	0.764
*P*	<0.001			0.168			
Total	476(50.7)	330(35.1)	133(14.2)	331(68.2)	147(30.3)	7(1.4)	<0.001

## Discussion

This study revealed a substantial decrease in prescription of single and multiple antimicrobials, as well as intravenous administration, in 2021 as compared with 2016. This may be attributed partly to the series of efforts taken to curb excessive antibiotic use made in the past 10–15 years in the study province. Although there was some evidence of reductions in antibiotics usage following these initiatives at county and tertiary-level hospitals by 2016 [24], our earlier study found little improvement at primary care settings, particularly in resource-poor rural areas [10]. Nevertheless, the data from 2021 suggest that national efforts to optimise antibiotics use have extended to the lowest level of hospital care. The outbreak of COVID-19 may also have contributed, to some extent, to the reduction of antibiotics use. Although Anhui province had found only 991 COVID-19 cases in total with no new cases for over a year by the time of the second survey was conducted [25], the epidemic introduced strict infection control measures, nationally and locally, through strengthening of healthcare reporting and monitoring, which together may have impacted positively on antimicrobial prescribing practice. Nevertheless, the rate of antibiotic prescriptions for symptomatic RTI patients from our 2021 study was still quite high at 70%, and contrasts with published rates at primary care settings in European countries for RTIs ranging from 10% to 52% [26, 27].

Our study also revealed a consistent decrease in the NDOV index value in all subgroups in 2021 from 2016. This may be due partly to China's proactive education campaign against COVID-19 in the past 2 years which urges residents to be highly vigilant to the disease, and to consult a doctor ‘at the first time’ when any related symptoms occur [28]. This appears to contradict a proposed strategy to contain bacterial resistance through delaying antimicrobial usage among RTI patients [19, 22]. As found in our earlier study [20], the trajectories of common RTI symptoms all feature a skewed peak, i.e. the frequency of symptom occurrence increased rapidly at the beginning, reached a sharp peak on day 1 or 2 following onset of infection and then tailed off over 15 days; this pattern mirrored the patients' intention to seek professional healthcare. More specifically, the chances for RTI patients to visit clinics and thus get prescribed antibiotics reduce sharply after their symptoms have peaked, i.e. 2–3 days following onset of infection. Therefore, China's future education of the public about COVID-19 merits closer scrutiny. In particular, community residents should be advised on the onset of COVID-19 or RTI-related symptoms [29–32], to seek immediate COVID-19 screening from a designated laboratory rather than medical care from a doctor, practice social distancing and wait for a few days to observe self-relief of the symptoms.

The differences found in the overall number and socio-demographic profiles of the participants in 2016 and 2021 may be explained largely by changes caused by the COVID-19 pandemic which led to widespread practices of social distancing, mask wearing and hand washing, etc. These measures not only helped in preventing COVID-19 infections but also other respiratory infections and contributed to the marked reduction (1072 *vs.* 702) in the total number of RTI patients recruited within the same length of study period and from the same number of site clinics. In addition to health, the pandemic had affected almost all commercial activities in China especially the service industry, e.g. restaurant, hotel, housekeeping and tourism. This may have contributed to a disproportional reduction in opportunities for female and older rural residents to find jobs in cities and who thus remained in the rural communities at the height of the pandemic in the 2021 survey [33, 34]. As for the disproportional changes in patients with upper *vs.* lower RTI, this could be attributed to increased awareness of upper respiratory infections because of the COVID-19 campaign. Patients with lower pulmonary infections, such as chronic bronchitis, generally have repeated experiences of their conditions and thus may be less likely to view their symptoms as COVID-19.

This study has both strengths and limitations. It compares data about service and antibiotics use collected at a 5-year interval in a single province using comparable sampling and survey approaches and revealed meaningful differences between the two study periods. Of note, the study was based on direct clinic observations which provided a valid assessment of antibiotics prescribing in rural healthcare facilities where record-keeping practices were not standardised across all sites as noted by others [35]. However, due to its descriptive nature, no adjustment for confounding factors was made in the analyses and diagnosis of RTI was based solely on clinical signs and symptoms. Consequently, it was not possible to determine the therapeutic appropriateness of prescribed antibiotics for the cause of infection. Moreover, direct observation may have influenced practice behaviours, as doctors may be more compliant to authorised guidelines when aware of being observed. Under the impact of the epidemic, the unemployment rate of migrant workers rose from 2.15% in 2018 to 3.63% in 2020 with a greater proportion of females to male (4.4% and 2.9%) respectively; likewise, the unemployment rate of newer migrant, and possibly younger workers was slightly lower than among older workers (3.4% and 4.0%, respectively) [36]. Seasonal differences in the prevalence of RTIs may also have impacted on the observed rates in the two study periods which took place in January and May – winter and spring – given the higher incidence of such infections in the former. Finally, although RTIs accounted for most medical consultations, they may not alone be reflective of all clinic attendances for which antibiotics were prescribed.

## Data Availability

The datasets generated and analysed during the study are not publicly available due to their potential use to identify participants. However, the datasets are available from the corresponding author on reasonable request.
